# Current Update on DWI-MRI and Its Radiomics in Liver Fibrosis—A Review of the Literature

**DOI:** 10.3390/tomography11060063

**Published:** 2025-05-30

**Authors:** Ali S. Alyami

**Affiliations:** Department of Diagnostic Radiography Technology, College of Nursing and Health Sciences, Jazan University, Jazan 45142, Saudi Arabia; aalmansour@jazanu.edu.sa

**Keywords:** liver fibrosis, assessment, DWI, IVIM-DWI

## Abstract

Introduction: Diffusion-weighted imaging (DWI) is a non-invasive technique for acquiring liver pathology data and characterizing liver lesions. This modality shows promise for applications in the initial diagnosis and monitoring of liver diseases, providing valuable insights for clinical assessment and treatment strategies. Intravoxel incoherent motion (IVIM), diffusion kurtosis imaging (DKI), and diffusion tensor imaging (DTI) are advanced forms of DWI. These techniques have proven effective for assessing liver lesions, including liver tumors and fibrosis. However, the results can be inconsistent. Thus, it is essential to summarize the current applications of these methods in liver fibrosis, identify existing limitations, and suggest future directions for development. Methods: This review assessed studies concerning liver DWI and its applications published in the PubMed database over the last nine years. It presents these techniques’ fundamental principles and key factors before discussing their application in liver fibrosis. Results and conclusions: It has been observed that advanced DWI sequences remain unreliable in ensuring the robustness and reproducibility of measurements when assessing liver fibrosis grades, due to inconsistent results and significant overlap among these techniques across different stages of fibrotic conditions.

## 1. Introduction

Liver fibrosis is a defining characteristic of chronic liver disease, characterized by excessive accumulation and disorganized distribution of extracellular matrix (ECM) proteins such as proteoglycans and collagen. These components, along with the non-collagenous elements of liver fibrosis, often contribute to hepatic dysfunction, the development of portal hypertension, progression to cirrhosis, and, in advanced cases, hepatocellular carcinoma [1]. Liver fibrosis is a dynamic process that occurs when inflammatory stimuli cause different cellular mediators to disrupt hepatic homeostasis. It constitutes one of the most common pathological manifestations of chronic hepatic disease, which is increasingly recognized as a significant global public health issue. Liver fibrosis is closely connected with substantial clinical morbidity and mortality, resulting in a considerable burden of disability and increased utilization of healthcare resources. This process leads to alterations in tissue architecture and is a defining characteristic of liver injury. Fortunately, liver fibrosis can be reversible, and there is potential for nearly complete recovery when the active injury is properly treated, especially in the early stages of fibrosis. Advanced liver fibrosis and cirrhosis are key risk factors for developing hepatocellular carcinoma [2]. Thus, the precise quantification of liver fibrosis is crucial for assessing the progression to liver cirrhosis, assessing the effectiveness of novel therapies, and determining the essential treatments, all of which significantly influence the prognosis of individuals with chronic liver diseases.

Different techniques can be used to evaluate liver fibrosis, including invasive and non-invasive approaches. Liver biopsy, an invasive technique, remains the gold standard for detecting and staging liver fibrosis. Histologic scoring systems classify the severity of fibrosis into various stages. For instance, the METAVIR system uses a semi-quantitative scoring approach, categorizing fibrosis as F0 (no fibrosis), F1 (minimal fibrosis, portal fibrosis without septa), F2 (moderate fibrosis, portal fibrosis with few septa), F3 (severe fibrosis, sepal fibrosis with many septa but no cirrhosis), and F4 (cirrhosis) [3]. Percutaneous liver biopsy, while diagnostically valuable, is inherently limited by sampling variability and carries non-negligible risks of major complications due to its invasive nature [4]. Therefore, there is a need for precise noninvasive examinations to detect and classify the stages of fibrosis accurately.

Medical imaging, such as elastography techniques using magnetic resonance (MRE) and ultrasound (USE), has increasingly gained attention. These imaging techniques represent the most accurate noninvasive modalities for evaluating liver fibrosis. However, these techniques have several limitations and are not widely accessible, so diagnostic tools are still needed for a reliable assessment of liver fibrosis. Furthermore, while conventional MRI sequences readily capture the morphological attributes of advanced liver fibrosis, they lack the sensitivity and specificity required to identify the early stages of fibrosis [5].

Quantitative magnetic resonance imaging (MRI) sequences, including MR elastography (MRE) and diffusion-weighted imaging (DWI), intravoxel incoherent motion (IVIM) sequences, have demonstrated promising effectiveness in assessing the degree of fibrotic changes in various anatomical regions. Moreover, these sequences are easily repeatable and do not require an intravenous contrast medium. DWI can capture data about Brownian motion, representing the stochastic motion of water molecules, and quantitatively indicate the degree of extracellular matrix accumulation using the apparent diffusion coefficient (ADC). This metric has been acknowledged as a dependable diagnostic instrument for liver fibrosis assessment [6]. DWI is effective for evaluating cirrhosis and liver fibrosis, as well as for detecting and staging liver metastatic tumors [7,8].

Radiomics represents an advanced computational methodology that utilizes standardized image processing techniques to derive high-dimensional quantitative features from medical imaging data, subsequently applying machine learning approaches for clinical decision support [9]. Numerous studies have thoroughly examined the role of radiomics in diagnosing and staging various types of cancer [10,11]. In addition to different clinical applications, radiomics has proven to be a valuable tool in assessing cardiomyopathy [12], musculoskeletal disorders [13], Crohn’s disease [14], and liver diseases. Quantitative imaging biomarkers extracted through radiomic analysis may improve diagnostic and prognostic clinical decision-making. For example, entropy-based parameters have demonstrated efficacy in differentiating malignant from benign cervical lesions [15]; a similar conclusion was drawn by Corino et al., who demonstrated the viability of utilizing DWI-based radiomics features for grading of soft tissue sarcomas [16]. When combined with radiomics, a quantitative technique designed to extract texture features from medical imaging that are not discernible to the human eye, this approach shows considerable potential for improving the assessment of liver fibrosis using MRI scans [17]. However, due to limited data, the diagnostic value of DWI for evaluating liver fibrosis remains unclear. Therefore, this review aims to consolidate the role of DWI and its advanced techniques in assessing liver fibrosis, providing a detailed overview of current DWI-radiomics applications and analyzing the accuracy of this technique for the automated diagnosis of liver fibrosis.

## 2. Methods

A thorough literature search of the Ovid MEDLINE and EMBASE databases identified pertinent publications, encompassing literature from 2016 through March 2025. The following keywords and search strategies were used: “Diffusion-weighted imaging or DWI or Intravoxel incoherent motion or IVIM” AND “hepatic/liver fibrosis”. “Diffusion kurtosis imaging or DKI or Diffusion tensor imaging or DTI” AND “hepatic/liver fibrosis”. For the radiomic section, the following words were used: DWI or DTI or DKI or IVIM and Radiomics AND liver fibrosis OR hepatic fibrosis. The search was restricted to articles published in English. This review included prospectively or retrospectively recruited patients with chronic liver fibrosis and performed radiomic analyses based on DWI-MRI or IVIM, DIT, or DKI to diagnose and/or differentiate liver fibrosis.

## 3. Assessment Techniques for Liver Fibrosis

Liver biopsy continues to be the definitive method for evaluating the extent of liver fibrosis and inflammation, maintaining a critical role in clinical diagnostics. Despite its diagnostic accuracy, the procedure is invasive and carries potential risks, often leading to patient reluctance and restricting its broader application in clinical settings. These limitations can delay critical therapeutic interventions, affecting patient outcomes. Furthermore, the reliability of pathological assessments may be compromised due to the potential for subjective interpretation by pathologists, particularly when the biopsy sample represents only a small portion of the liver. Such constraints can reduce the precision of diagnostic evaluations, ultimately influencing liver fibrosis’s comprehensive assessment and management [18].

Various scoring systems have been established for histopathologically assessing chronic liver disease in recent years. The Kleiner scoring system is utilized for individuals with steatotic liver disease and consists of five primary groups (0 to 4) that assess liver fibrosis. Notably, stage 1 is subdivided into three distinct subcategories [19]. The Ishak scoring system offers a more detailed and nuanced classification, encompassing seven distinct grades that reflect the extent and distribution of fibrotic changes and liver architecture alterations [20]. The METAVIR scoring system is widely recognized as a benchmark for assessing chronic hepatitis C, offering a standardized framework that classifies necro-inflammatory activity into four levels and liver fibrosis into five distinct stages [21]. A summary of these scores can be found in Table 1.

Noninvasive methods for evaluating liver fibrosis have become essential components of routine clinical practice for individuals with suspected or diagnosed liver conditions. A range of biomarkers are employed to assess liver fibrosis, including specialized markers associated with fibrosis (e.g., laminin and hyaluronic acid) and serological parameters (e.g., platelet counts and liver enzyme levels) [22]. Although variations in these biomarkers can provide valuable information about the progression of liver fibrosis, their accuracy and specificity are often limited by various confounding factors. This complicates the precise determination of the actual extent of fibrotic changes in the liver.

## 4. Basic Principles of DWI and Its Advancements

DWI is an MRI sequence that quantitatively assesses the Brownian motion of water molecules within tissues, which is limited in fibrosis due to the formation of fibrous septa and collagen deposition. There are two models used to analyze the data obtained from DWI sequences: the monoexponential model, which necessitates at least two b-values: 0 and ≤200 s/mm^2^ to calculate the ADC, and the bi-exponential model, employed in a technique known as IVIM, which requires multiple b-values to generate several DWI images. DWI provides qualitative and quantitative insights into the microarchitectural and microperfusional characteristics of tissue structure without needing a contrast agent [23].

**Figure 1:**
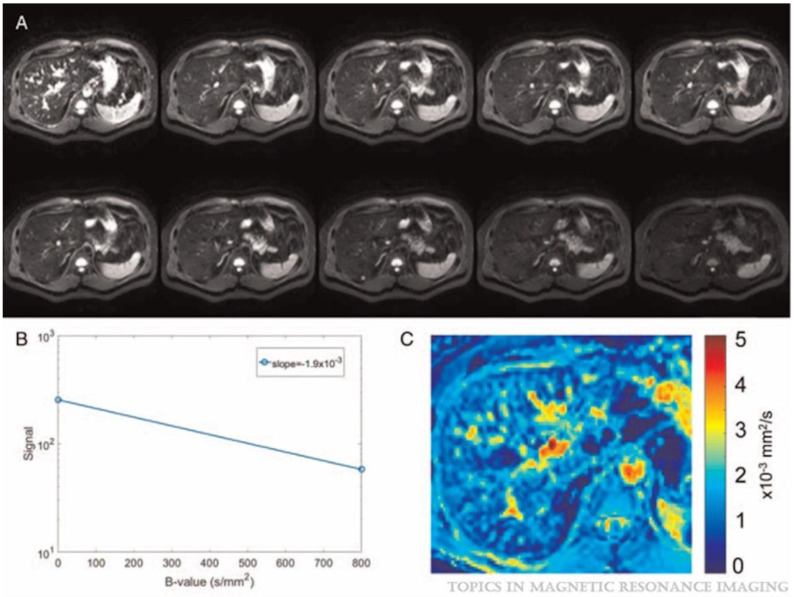
DWI acquired (**A**) with 10 B values (b = 0, 10, 20, 30, 40, 50, 100, 200, 400, and 800 s/mm^2^). (**B**) The signal from the two extreme b-values is plotted semi-logarithmically as a function of their b value and a linear regression is used to extract the ADC (mm^2^/s) for a specific pixel. (**C**) A color parametric map of ADC values is generated for each pixel in the image [8].

To create ADC maps, measurements are typically collected using two b-values (often three). For each voxel in the MRI scan, the logarithm of the measured signal is plotted against the applied b-values. The resulting linear relationship allows the ADC to be determined from the slope of this plot, as described by Equation (1):

(1) S(b)=S0exp⁡(−ADC.b)

where S0 indicates the mean signal intensity at b = 0 s/mm^2^ and S(b) refers to the mean signal intensity at specific b values. ADC is widely utilized as a non-invasive diagnostic tool for evaluating liver fibrosis. It can be extracted using a mono-exponential model as seen in Figure 1. This figure was taken from Figure 3 in Petitclerc et al.’s paper [8].

**Figure 2:**
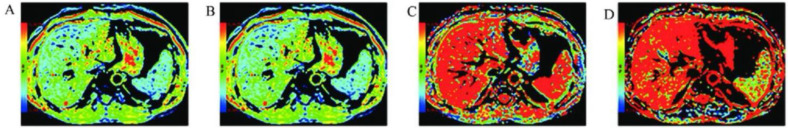
58-year-old man with mild fibrosis (stage 1) and hepatitis (grade 1). Illustration of (**A**) apparent diffusion coefficient; (**B**) real diffusion coefficient; (**C**) pseudo-diffusion coefficient; and (**D**) perfusion fraction. This figure was taken from Figure 1 in Lesheng Huang et al.’s study [27].

IVIM is a bi-exponential model of DWI introduced by Le Bihan et al. [24], enabling the separate evaluation of microcirculation in capillaries and the diffusion of water molecules. This technique, classified as one of the widely non-Gaussian models, fits multiple b-values DWI data to a bi-exponential decay equation to separate diffusion from perfusion. In the IVIM model, the molecular perfusion with high b values (> or equal to 200 s/mm^2^) and the capillary perfusion could be evaluated with low b values (<200 s/mm^2^). This model can obtain different parameters, such as true diffusion, which depends on Gaussian diffusion, whereas pseudo-diffusion (perfusion) is caused by blood movement in capillaries. According to the IVIM framework, the related parameters include a fast diffusion compartment associated with microperfusion (Dfast or D*), the perfusion fraction (PF or f), and a slow compartment linked to pure molecular diffusion (Dslow or D) [25]. The biexponential model of IVIM can be expressed using Equation (2):

(2) $$SI/{SI}_{0}=(1-PF)exp\left(-b.D\right)+PFexp(-b.D^{*})$$

where SI0 and SI are the signal intensity at b = 0 s/mm^2^ and given b-values, respectively; b is the b-factor, D* is the perfusion coefficient, D is the true diffusion coefficient, and PF is the fractional perfusion [26]. The DWI and IVIM parameters are presented in Figure 2. This figure was taken from Figure 1 in Huang et al.’s study [27].

The stretched-exponential model is an advanced model of IVIM that characterizes the non-Gaussian diffusion behavior of data acquired at b-values between 1000 and 3000 s/mm^2^. In this model, the stretching exponent, γ, is associated with the heterogeneity of the medium through which the spins diffuse. The stretched exponential model’s distributed diffusion coefficient (DDC) allowed for the exact and precise staging of liver fibrosis, regardless of the presence of steatosis [28,29].

Diffusion kurtosis imaging (DKI) is another advanced sequence of DWI. Jensen et al. developed this tool for estimating the extent of water diffusion in biological tissues, utilizing a non-Gaussian distribution model in 2005 [30]. Kurtosis is defined as the standardized and normalized fourth central moment of the distribution of water displacement. In a modified image post-processing procedure, higher b-values are computed to derive quantitative insights into the magnitude and direction of non-Gaussian water diffusion displacement, which includes mean kurtosis (MK) and mean diffusivity (MD) [30,31]. An increased kurtosis value indicates a more constrained diffusion environment.

Diffusion tensor imaging (DTI) is another advanced MRI sequence that has recently been developed, building upon the principles of DWI. This method allows the characterization of the magnitude and orientation of the anisotropic diffusion. DTI measures the diffusivity of water molecules using six or more different directions of diffusion-sensitive gradients, in contrast to unidirectional or three orthogonal directional DWI. This method distinctly illustrates the microstructural features of biological tissues, enabling the assessment of their anisotropic properties [32]. The DTI sequence generates parametric maps, including average diffusion coefficient, fractional anisotropy (FA), and relative anisotropy images, alongside their respective quantitative metrics. DTI does not require contrast agents, works on most modern MRI machines, and the scan itself does not take long to perform. Table 2 compares the advantages and disadvantages of different DWI techniques in liver disease.

## 5. The Principal Changes in Hepatic Fibrosis Molecules Using DWI

The DWI sequence is based on the Brownian motion of water molecules in specific tissues. Conventional monoexponential DWI indirectly identifies tissue microstructural alterations by quantifying the direction and extent of water molecule diffusion to determine the ADC [33]. In liver fibrosis, cellular membranes and collagen fibers can impede the diffusion of water molecules, leading to alterations in signal intensity and ADC values [34]. In general, fibrosis results in decreased mobility of water molecules, leading to a reduction in ADC values. Consequently, tissues with greater fibrosis typically exhibit lower ADC values on DWI. Thus, ADC values may serve as a potential biomarker for evaluating the extent of fibrosis in various tissues, including the liver.

Several studies have been conducted to describe the utilization of DWI as a complementary technique to conventional MRI for evaluating the degree of liver fibrosis [35,36]. A retrospective study was conducted to validate DWI-based virtual elastography in diagnosing liver fibrosis [35]. Researchers extracted the ADC from DWI data and turned it into the DWI MRI-based virtual shear modulus, µDiff, demonstrating significant agreement with µMRE. These findings indicate a potential direction for future virtual elastography techniques that eliminate the need for supplementary mechanical vibration apparatus. In a single prospective study conducted by Jiaoyan Wang et al. [36] to establish the optimal high and low b-values of DWI for assessing hepatic fibrosis in 81 patients with chronic liver disease and 21 healthy volunteers. Twenty combinations of nine b-values: 0, 50, 100, 150, 200, 800, 1000, 1200, and 1500 s/mm^2^ were used to calculate the ADC. Liver biopsy was used as a gold standard. The investigators found that all the ADC values within the cohort of healthy volunteers exhibited a statistically significant increase compared to those recorded in the group diagnosed with hepatic fibrosis (all *p* < 0.01). When differentiating between the stages of liver fibrosis, ADC values derived from b-values of 200 and 800 s/mm^2^ and 200 and 1000 s/mm^2^ were shown to be more effective. As the advancement of liver fibrosis occurred, there was a notable and statistically significant decrease observed in the ADC values across the b-value pairings of 100 and 1000 s/mm^2^, 150 and 1200 s/mm^2^, 200 and 800 s/mm^2^, as well as 200 and 1000 s/mm^2^. No significant differences were observed between the ADC values and the stages of fibrosis for the following b-value combinations: 0 and 1500 s/mm^2^, 50 and 800 s/mm^2^, 50 and 1000 s/mm^2^, 50 and 1500 s/mm^2^, 100 and 800 s/mm^2^, 100 and 1500 s/mm^2^, 150 and 1000 s/mm^2^, and 150 and 1500 s/mm^2^, with all *p*-values > 0.01. Moreover, the correlations between ADC and MRE shear stiffness values obtained from DWI were statistically significant for all the b-value combinations across all the subjects (all *p* < 0.001). They concluded that the two combinations of b values of 200 and 1000 s/mm^2^ and 200 and 800 s/mm^2^ were recommended to identify the stages of hepatic fibrosis.

DWI has been utilized to quantify liver fibrosis noninvasively; however, it is inadequate for distinguishing between different stages of hepatic fibrosis. DKI and IVIM provide a more accurate representation of the variations in non-Gaussian water diffusion observed in DWI. Several studies have been conducted using these techniques to evaluate liver fibrosis [28,37,38,39,40]. Lesheng Huang et al. [27] evaluated the diagnostic effectiveness of metrics derived from DKI, IVIM, and DWI to stage liver fibrosis, focusing on the foundation of robust inter-examiner reliability. The study included 145 subjects (48 healthy volunteers, 59 early liver fibrosis patients, and 38 advanced liver fibrosis patients). A biopsy was used to confirm liver fibrosis. Two assessors analyzed IVIM using DKI and IVIM-DWI models. Parameters from DTI, DKI, and IVIM-DWI with an intraclass correlation coefficient ICC equal to or greater than 0.6 were used to build regression models for healthy volunteers vs. early liver fibrosis and early vs. advanced liver fibrosis. The inter-examiner reliability demonstrated excellence in the IVIM-DWI parameter variations in the rates of intravoxel water diffusion represented α (ICC: 0.81–0.84), for the DTI parameters fractional anisotropy (FA) (ICC: 0.83–0.91), radial diffusivity (RD) (ICC: 0.86–0.96), axial diffusivity (AD) (ICC: 0.80–0.97), and MD (ICC: 0.80–0.96); and the DKI parameters radial kurtosis (RK) (ICC: 0.88–0.95), axial kurtosis (AK) (ICC: 0.86–0.96), and MK (ICC: 0.91–0.98). Moreover, the inter-examiner reliability was good for the DWI-derived ADC (ICC: 0.69–0.83), IVIM-DWI-derived perfusion fraction (f) (ICC: 0.71–0.79), and the DKI-derived fractional anisotropy of kurtosis (FKA) (ICC: 0.75–0.92). However, a moderate-to-poor inter-examiner reliability was observed for the DWI-IVIM parameter’s DDC (ICC: 0.58–0.67), pseudo-diffusion coefficient (D*) (ICC: 0.27–0.76), and real diffusion coefficient (D) (ICC: 0.58–0.67).

Moreover, DWI, especially the IVIM technique, has the potential to categorize individuals with viral hepatitis based on their fibrotic status [39,41], with recent studies successfully differentiating between cirrhotic and healthy livers [42,43,44] and classifying the severity of the disease using scoring systems based on blood biomarkers [45]. A prospective study aimed to assess the role of the IVIM diffusion model in assessing liver inflammation and fibrosis in diffuse liver conditions while also taking into account the existence of liver steatosis and iron deposits. The study included 42 healthy volunteers and 54 individuals diagnosed with chronic hepatitis B. Sixteen b values were used, and IVIM parameters f, D*, and D were calculated. The histological examination of biopsy samples was used as the gold standard for the staging of liver inflammation and fibrosis. With a Cohen’s d effect size of 0.452, the patient group’s D values were significantly lower than those of the control group (*p* = 0.038). There was a significant difference in D values across the various stages of fibrosis, with D values decreasing from minimal to moderate to marked fibrosis. The degree of fibrosis showed a statistically significant inverse correlation with D* and D values (*p* = 0.021 and *p* = 0.001, respectively). Furthermore, inflammation grades showed a negative correlation with f values (*p* = 0.047). They concluded that D values obtained from IVIM imaging may assist in diagnosing liver fibrosis [43]. A previous study suggested that IVIM parameters can be used to separate healthy livers from fibrotic livers [44]. Wáng and colleagues studied 33 hepatitis-b liver fibrosis patients and healthy volunteers. The findings of Wáng and colleagues were particularly significant in distinguishing between patients without liver fibrosis and those with F1 or F2 fibrosis in cases of viral hepatitis, demonstrating an AUC of 0.986 and 1, respectively. They also reported that a b-value threshold of 60 s/mm^2^ was preferred over 200 s/mm^2^ for distinguishing between healthy volunteers and individuals with liver fibrosis.

In another retrospective study, Park et al. [29] evaluated the diagnostic performance of the stretched exponential DWI model in comparison to both mono- and biexponential DWI models and transient elastography, as well as to assess the impact of hepatic inflammation and steatosis on these DWI models and transient elastography. Pathological evaluation served as the gold standard for reference. The study findings indicated that the DDC derived from the stretched exponential DWI model exhibited the highest diagnostic performance for staging liver fibrosis, followed closely by transient elastography; however, there was no statistically significant difference between these two methods. Additionally, only DDC was able to distinguish between F0–1 and F2–3 as well as between F2–3 and F4 stages. Fibrosis was found to be the only independent factor influencing DDC and transient elastography, although both DDC and transient elastography demonstrated strong associations with hepatic inflammation and fibrosis. Furthermore, there was no association with the stretched exponential DWI or transient elastography, but there were associations with the ADC, pseudo-diffusion coefficient, and true diffusion coefficient.

Hu et al. [46] conducted a retrospective study to assess the feasibility of IVIM-DWI imaging through histogram analysis for liver fibrosis staging. The study relied on histopathologic results as a gold standard and involved 56 individuals diagnosed with chronic liver disease. Nine b-values 0, 25, 50, 75, 100, 150, 200, 500, and 800 s/mm^2^ and the METAVIR scoring system were used to determine the fibrosis level. Participants were classified into three groups according to METAVIR fibrosis staging: F0–1 (*n* = 25), F2–3 (*n* = 21), and F4 (*n* = 10). There were statistically significant variances in the mean, interquartile range, and percentiles (50th, 75th, and 90th) of D* maps among the groups (all *p* < 0.05). The AUC values for the mean, interquartile range, and the 50th, 75th, and 90th percentiles of D* maps in detecting significant liver fibrosis (≥F2 stage) were 0.901, 0.859, 0.876, 0.943, and 0.886, respectively (all *p* < 0.0001); whereas, for diagnosing severe fibrosis (F4), the AUC values were 0.917, 0.922, 0.943, 0.985, and 0.939, respectively (all *p* < 0.0001). There were no significant differences among the other histogram metrics ADC, D, and f maps among all the groups (all *p* > 0.05). A recent retrospective study conducted by Ren et al., involving 106 patients, revealed a moderate association between pseudo-diffusion and perfusion fractions across varying fibrosis stages [47].

Stretched exponential models and DKI are based on a non-Gaussian diffusion distribution, offering supplemental insights and serving as valuable instruments for the characterization of liver fibrosis. Few studies on liver fibrosis using DKI have yielded inconsistent results [40,48]. In a prospective study, Li Yang [40] compared the efficacy of DKI and conventional DWI in staging liver fibrosis among 81 individuals with chronic liver disease, with histopathological analysis as the gold standard. Nine b-values of 0, 200, 500, 1000, 1500, and 2000 s/mm^2^ were used in this study. Different parameters were calculated, including ADC maps, MK, and MD. The study evaluated the diagnostic performance of quantitative metrics for identifying ≥F2 and ≥F3 fibrosis stages. Statistical analysis revealed significant correlations between fibrosis severity and ADC (rho = −0.496), MK (rho = 0.537), and MD (rho = −0.491) (all *p*-values < 0.001). Notably, ADC demonstrated a moderate positive association with MD (rho = 0.601) and a near-perfect inverse relationship with MK (rho = −0.968) (all *p*-values < 0.001). No significant differences (*p* > 0.05) were observed in the discriminative ability for ≥F2 fibrosis among the evaluated parameters: ADC (AUC = 0.797), MK (AUC = 0.809), and MD (AUC = 0.715). Similarly, AUC values were comparable for ADC (0.747), MK (0.768), and MD (0.710) in predicting ≥F3. Recent studies by Fu et al. [49] and Seo et al. [28] have highlighted the superior diagnostic capability of the DDC derived from the stretched exponential model, particularly in staging liver fibrosis at levels greater than F2, when compared to DWI and IVIM. The findings of Fu et al. [49] study reported that the stages of liver fibrosis, as determined by pathology, have been found to have a negative correlation with both D slow and f. Moreover, Seo et al. [28] concluded that stretched exponential DWI represents a promising technique, demonstrating strong diagnostic capabilities while requiring fewer b-value acquisitions, thereby facilitating reduced acquisition times.

Yoshimaru et al. [48] evaluated the ability of DKI analysis using the breath-hold strategy to evaluate and classify liver fibrosis in 67 patients. Different parameters were used to assess liver fibrosis, including MK, MD, and ADC values among patients with varying levels of fibrosis: F0–F1, F2–F3, and F4. There was a significant variation in the ADC, MD, and MK values between the non-cirrhosis and cirrhosis groups. There were no significant differences in ADC values across all groups (all *p* > 0.05). However, statistically significant differences in the MD values were observed between the F0–F1 and F4 groups, as well as between the F2–F3 and F4 groups, with all *p*-values < 0.05. Furthermore, statistically significant differences were found in the MK values between the F0–F1 and F2–F3 groups, the F0–F1 and F4 groups, and the F2–F3 and F4 groups, with all *p*-values being <0.05. No correlation was found between the degree of liver fibrosis staging and ADC values (Spearman’s rank correlation coefficient ρ = −0.227; *p* = 0.078). However, there was a significant correlation between MD and MK values with the extent of liver fibrosis staging (ρ = 0.851 and −0.672; *p* < 0.0001). Following ROC analysis to assess various fibrosis stages including F0, fibrosis (≥F1), advanced fibrosis (≥F2), and liver cirrhosis, the DKI cutoff values were established as 0.923, 0.955, and 1.11, respectively.

A prospective study compared the diagnostic accuracy of liver conventional DWI and DKI in differentiating healthy individuals (*n* = 27) from patients (*n* = 45) with mild and substantial fibrosis [50]. A liver biopsy was used to recruit the study participants. Statistically significant variances in ADC and MD values were observed among the groups (*p* < 0.05). A significant difference in ADC values was observed when comparing S0 and S2 groups (*p* = 0.012), indicating discriminative capability between these fibrosis stages. They found that the MD values decreased as the fibrosis advanced. Specifically, the MD value exhibited statistical distinctions between S0 and S1 (*p* = 0.028) and S0 and S2 (*p* = 0.005). The MK value showed no significant differences across the groups (*p* = 0.646). Significant correlations were observed between MD and ADC values with the stages of fibrosis (rs = −0.668, −0.341; *p* < 0.01). In contrast, no significant correlation was detected between MK values and fibrosis stages (rs = 0.180; *p* = 0.130). The AUC values for ADC and MD were 0.707 and 0.937, respectively, for distinguishing between stages S1–2, and 0.817 and 0.658 for stage S2. MD demonstrated superior performance compared to ADC in characterizing S1–2 and S2 (*p* < 0.05). Ren et al. [51] assessed the clinical utility of multi-modal DWI for evaluating liver fibrosis. Their findings suggested that the IVIM-D* value derived from the bi-exponential model, along with the SEM-DDC obtained from the SEM model, demonstrated superior performance compared to other parameters in assessing the severity of liver fibrosis.

In a recent prospective investigation, 17 individuals without known liver conditions and 59 patients with suspected liver disease scheduled for liver biopsy were included to evaluate the clinical utility of six distinct DWI models for staging liver fibrosis and to compare their diagnostic efficacy [52]. These models included ADC, IVIM-D, and DKI-MD. Several b-values DWI were used: 0, 50, 100, 150, 200, 400, 600, 800, 1000, 1200, 1500, and 2000 s/mm^2^. A notable association was observed between ADC, IVIM-D, and DKI-MD values and the fibrosis stages. A compilation of reports on DWI for assessing liver fibrosis has been organized and summarized in Table 3.

## 6. Radiomics Analysis of DWI Images in Liver Fibrosis

Radiomics is a post-radiology process that involves the high-throughput extraction of features from radiological images, transforming them into mineable data. This process incorporates sophisticated artificial intelligence techniques, including convolutional neural networks, deep learning, and machine learning to enhance predictive accuracy. Radiomics is an emerging field in the pursuit of precision medicine, aiming to develop novel biomarkers and offer deeper, subvisual insights into the tumor microenvironment, immunophenotype, and tissue pathology. This field employs semi-automated or automated quantitative analyses of high-dimensional images to improve medical disorders’ diagnosis, characterization, and prognosis. It has received increasing attention over the past decade [54].

Liver fibrosis, along with its advanced stage of cirrhosis, represents a progressive disease process that poses significant clinical challenges, notably an increased likelihood of developing portal hypertension and hepatocellular carcinoma. The integration of radiomics, a quantitative analysis method aimed at extracting texture features from medical imaging that are not readily observable, presents considerable potential for improving the assessment of liver fibrosis through MRI exams [17,55]. Considering the heterogeneity in liver fibrosis and the possible sampling errors related to liver biopsies, radiomics analysis of a region of interest that includes the whole liver parenchyma might better assess liver fibrosis than standard biopsy techniques.

In a prospective single-center study, Qiu et al. [56] formulated a radiomics model aimed at staging liver fibrosis and precisely identifying early-stage cirrhosis. This study utilized a feature extraction technique applied to DWI-MR images from 369 patients, which included 145 individuals with healthy livers, 116 with early-stage cirrhosis, and 108 with liver fibrosis. This study used three b-values: 0, 400, and 800 s/mm^2^. Two experienced abdominal radiologists delineated the regions of liver fibrosis and early-stage cirrhosis. For maximal accuracy, the classification model was developed using two distinct approaches, each comprising two separate models. Plan 1, Model 1, was initially developed to differentiate between abnormal and healthy livers. Following this, Model 2 was constructed based on the output of Model 1 to distinguish between liver fibrosis and early-stage cirrhosis within the abnormal liver category. In Plan 2, Models 1 and 2 were developed simultaneously to classify healthy liver versus liver fibrosis and healthy liver versus early-stage cirrhosis, respectively. The study included different machine learning algorithms that were employed to train and validate the classification models based on the extracted features, such as univariate analysis and RELIEFF feature selection. The classification model was constructed using the support vector machine algorithm with a radial basis function kernel. The research team reported that the best models were developed in Plan 1. For Model 1 in this approach, the area AUC values for the training and validation cohorts were 0.973 (95% confidence interval [CI] 0.946–1.000) and 0.948 (95% CI 0.903–0.993), respectively. For Model 2 in Plan 1, the area AUC values for the training and validation cohorts were 0.944 (95% CI 0.905–0.983) and 0.968 (95% CI 0.940–0.996), respectively. In Plan 1, Model 1, which utilized a support vector machine model to distinguish between healthy and abnormal liver conditions, attained an accuracy of 91.5% (95% CI, 89.3–93.7%) and a training cohort receiver operating characteristic AUC of 0.973 (95% CI, 0.946–1.000). In the validation cohort, the model demonstrated an accuracy of 89.1% (95% CI, 86.4–91.8%) and an AUC of 0.948 (95% CI, 0.903–0.993). In the training cohort, the support vector machine model for Model 2 in Plan 1, which was intended to detect liver fibrosis and early-stage cirrhosis, attained an accuracy of 88.9% (95% CI, 87.3–90.5%) and an AUC of 0.944 (95% CI, 0.905–0.983). The model’s accuracy and AUC in the validation cohort were 92.6% (95% CI, 90.4–94.8%) and 0.968 (95% CI, 0.940–0.996), respectively. The research team concluded that the radiomics analysis of DWI images demonstrated high accuracy in distinguishing between study categories, with robust statistical measures indicating its effectiveness.

In a recent study, Longyang Xiao et al. conducted a retrospective analysis aimed at developing and comparing a fusion model and a radiomics model, which integrates serum fibrosis biomarkers with radiomic features, utilizing multiple MRI parameters, including DWI, for the staging of liver fibrosis in patients with chronic liver disease [57]. Five different parametric MR images were used for building the radiomics model. Two hundred thirty patients were included in their study and divided into groups randomly divided into a testing group (*n* = 70) and a training group (*n* = 160) with a ratio of 3 to 7. Radiomics features with ICC > 0.8 were selected. From the DW image, 337 features were selected. To differentiate between F1–3 and F4, the model’s radiomics features included Wavelet HLH GLRLM-Long run low gray level emphasis (coefficient: 0.380) from DW images, Wavelet LLL GLRLM-Run entropy (coefficient: 0.746) from DW images, and Wavelet LHL GLCM-Correlation (coefficient: −0.421) from DW images. The model’s radiomics features, such as Wavelet HLL GLCM-Maximum probability (coefficient: −0.265) from DW images and Wavelet HLH GLCM-Maximum probability (coefficient: −0.307), were utilized to differentiate between F1–2 and F3. They found that the AUCs of the radiomics model for staging liver fibrosis ranged from 0.514 to 0.724 in the testing cohort and from 0.707 to 0.842 in the training cohort. Another study focused on a noninvasive method for grading liver fibrosis using texture analysis derived from MRI-based radiomics. By analyzing the subtle texture features in MRI scans, including ADC, Gotta et al. aimed to develop a reliable grading system for liver fibrosis that does not require invasive procedures [58]. A subanalysis investigating the effect of incorporating ADC into radiomics models for predicting fibrosis grades indicated a reduction in model performance, as evidenced by lower area AUC values relative to radiomics alone. In fibrosis grade 2, the radiomics model reached its highest area AUC at 0.830. However, when ADC was integrated into the model, the AUC diminished to 0.694. The inclusion of ADC only led to a modest enhancement in the model for fibrosis grade 3, raising the AUC from 0.610 to 0.700. Nonetheless, these variations in AUC values were not statistically significant (*p* > 0.05) [58]. Table 4 summarizes and organizes a compilation of radiomics reports to assess liver fibrosis.

## 7. Discussion

Liver fibrosis is characterized by the accumulation of ECM, including collagen, which hinders water movement within the liver. DWI is a commonly utilized MRI technique that does not require additional hardware or the use of contrast agents. DWI has been utilized to evaluate liver fibrosis. Studies have reported a significant reduction in ADC values corresponding to the progression of liver fibrosis and reported that the liver fibrosis group’s ADC value is lower than that in the normal control group [51]. Moreover, previous studies reported that comparing DWI and MRE for the staging of liver fibrosis have demonstrated that the diagnostic performance of DWI is inferior to that of MRE.

Most studies [37,44,53] found that liver fibrosis is associated with a decrease in all three IVIM parameters (Dslow, Dfast, and PF) as liver fibrosis increases. Among these parameters, PF has consistently demonstrated the greatest sensitivity to changes in liver fibrosis, likely because the estimation of the Dfast presents significant challenges. Conversely, the Dslow can exhibit a substantial decrease in certain cases of fibrotic liver [37,44,53]. Few studies [48,50] found that correlation analysis showed MD values were moderately associated with the fibrosis stages. However, these findings were higher than in another study [40].

## 8. Future Research Directions for DWI in Liver Fibrosis Assessment

The current limitations of DWI methods in evaluating liver fibrosis, including staging overlap, measurement variability, and reliance on post-processing methods, highlight the necessity for targeted future research. Regarding hardware and software development, high-field MRI systems (≥3 T) could greatly enhance the sensitivity of DWI for early fibrosis detection by providing improved spatial resolution and signal-to-noise ratios. For instance, a magnetic field of 3.0 Tesla demonstrates increased sensitivity to artifacts caused by magnetic susceptibility and eddy current distortions. Therefore, future research should concentrate on standardizing and optimizing IVIM protocols across different magnetic field strengths to improve clinical applications.

Future advancements in radiomics may transform liver disease assessment by implementing fully automated analysis platforms. Such systems could significantly enhance diagnostic workflows by eliminating manual processing steps, thereby reducing interobserver variability while improving both the efficiency and reproducibility of quantitative imaging evaluations. The integration of artificial intelligence-driven automation in radiomic analysis pipelines holds particular promise for standardizing fibrosis assessment across clinical settings, potentially enabling a more objective and high-throughput characterization of hepatic pathology. These technological developments could address current limitations in traditional radiomic approaches by providing consistent, rapid, and operator-independent analysis of DWI biomarkers for liver fibrosis staging. In addition, investigating underexplored domains, such as integrating radiomics with genomic data, presents significant opportunities for enhancing our comprehension of liver diseases, including liver fibrosis. This methodology may uncover relationships between DWI techniques and genetic factors.

## 9. Conclusions

Overall, different studies have confirmed the effectiveness of DWI in detecting significant liver fibrosis, establishing it as a valuable tool for diagnosing it. However, other diffusion models did not offer any further diagnostic value beyond the bi-exponential IVIM model or conventional DWI for identifying and staging liver fibrosis, except the stretched exponential model.

## Figures and Tables

**Table 1:** Example of Commonly Utilized Histopathological Scoring Systems for Grading Liver Fibrosis.

Fibrosis Stages	METAVIR System	Ishak System	Kleiner System
Stage: 0	Absence of fibrosis	No evidence of fibrosis	No fibrosis observed
Stage: 1	Fibrosis limited to portal areas without evidence of bridging septa	Mild fibrous expansion in some portal areas, accompanied by or without short septa	1: Fibrosis in periportal or presinusoidal regions
			1A: Zone 3 has mild perisinusoidal fibrosis
			1B: Zone 3 has moderate perisinusoidal fibrosis
			1C: Fibrosis in periportal or portal areas
Stage: 2	Portal fibrosis with minimal septa	Fibrous expansion in most portal areas, with or without short septa	Combined perisinusoidal and periportal/portal fibrosis
Stage: 3	Significant septal formation without cirrhosis	Fibrous expansion in majority portal regions, with occasional bridging	Presence of bridging fibrosis
Stage: 4	Development of cirrhosis	Extensive bridging fibrosis, including portal-to-central bridging and portal-to-portal	Cirrhosis
Stage: 5		Prominent bridging fibrosis with early nodule formation	
Stage: 6		Established cirrhosis	

**Table 2:** Comparison of DWI techniques for liver fibrosis assessment in terms of advantages and disadvantages.

Technique	Advantages	Disadvantages
Conventional DWI	- Non-invasive and widely available in most of MRI system - No contrast agent required - Simple post-processing (ADC calculation) - No need for additional hardware	- Insufficient for differentiating between liver fibrosis stages - ADC cannot accurately diagnose the duration of liver fibrosis - Overlap in ADC values across fibrosis stages - Technique reproducibility and standardization issue - Motion artifact, suboptimal fat suppression and iron deposition reduces the accuracy of the measurement
IVIM-DWI	- Separates perfusion (D* and f) and diffusion (D) components - Better sensitivity for early fibrosis than conventional DWI - No contrast agent needed	- Complex post-processing (requires multiple b-values) - Poor reproducibility for perfusion parameters (D*) - Limited standardization across studies
DKI	- Captures non-Gaussian diffusion, reflecting tissue complexity - Potential to differentiate fibrosis stages	- Requires high b-values (>1000 s/mm^2^), increasing scan time - Inconsistent diagnostic performance across studies - Limited clinical validation
DTI	- Assesses anisotropic diffusion (useful for fibrous tissue structure) - Provides directional diffusion metrics	- Long acquisition time (multiple directions) - Limited evidence for liver fibrosis staging - Sensitive to motion artifacts

**Table 3:** Summary of DWI studies to evaluate liver fibrosis.

Ref.	Target and Study Type	Aim	Results
Kromrey et al. [35]	N = 74 Retrospective	To assess the effectiveness of DW-MRI-based elastography for staging liver fibrosis	There was a strong agreement between the µDiff and µMRE values (mean difference of −0.02 kPa ± 0.88; *p* < 0.001). In 55% of patients, DWI-based fibrosis staging matched magnetic MRE staging, with a one-stage difference in 35% of cases.
Park et al. [29]	N = 87 Retrospective	To assess the diagnostic effectiveness of the stretched exponential model of DWI and to examine the impact of confounding factors on the staging of liver fibrosis	The DDC showed no correlation with steatosis (*p* = 0.619) but demonstrated a significant correlation with inflammation (*p* = 0.001) and fibrosis (*p* < 0.001). The DDC exhibited superior performance for liver fibrosis with an area under the curve of 0.717 (95% CI: 0.653–0.765) compared to transient elastography, which had a performance of 0.681 (95% CI: 0.623–0.733).
Jiaoyan Wang et al. [36]	110 subjects (21 healthy liver and 81 patients with liver fibrosis) Prospective	To identify the optimal high and low b-values of DWI for assessing hepatic fibrosis	The analysis revealed significant correlations between all tested b-value-derived ADC measurements and MRE values (all *p* < 0.05). Particularly strong associations were observed for three specific b-value ranges: 0–800 s/mm^2^, 200–1000 s/mm^2^, and 200–1200 s/mm^2^. The ADC with b-values of 200–800 s/mm^2^ and 200–1000 s/mm^2^ had area under the receiver operating characteristic (AUROC) values greater than 0.750 for detecting hepatic fibrosis in the F1 and F2–4 groups, F1–2 and F3–4 groups, and F1–3 and F4 groups, respectively. The b-value combination of 200–800 s/mm^2^ demonstrated superior diagnostic accuracy for fibrosis staging compared to the 200–1000 s/mm^2^.
Huang et al. [53]	Hepatitis-b induced liver fibrosis group (*n* = 12, F1–2 = 7, and F3–4 = 5) and non-fibrosis group (*n* = 30) Prospective	To assess the diagnostic effectiveness of the modified IVIM technique in detecting early-stage liver fibrosis	To differentiate between liver fibrosis patients and healthy volunteers, a threshold b-value of 60 s/mm^2^ was preferred above 200 s/mm^2^. When a threshold b-value of 60 s/mm^2^ was used, a PF (f) less than 6.49% effectively distinguished healthy livers from all fibrotic livers with 100% sensitivity and specificity. For the patient measurements, there was a strong correlation between PF and Dfast, with a Pearson correlation coefficient of r = 0.865 (*p* < 0.001). However, no significant correlation was observed between the slow diffusion component (Dslow) and the fast diffusion compartment (Dfast or PF). For Dslow, Dfast, and PF, the intraclass correlation coefficient (ICC) of intra-reader agreement was 0.909, 0.949, and 0.925, respectively.
Wáng et al. [44]	N = 49 subjects (33 hepatitis-b induced liver fibrosis and 16 healthy participants	To investigate a combined use of intravoxe IVIM parameters for liver fibrosis assessment	Compared to healthy volunteers, liver fibrosis had lower values in terms of PF, Dslow, and Dfast. Among these parameters, PF demonstrated the highest diagnostic value, followed by Dslow. The regression and classification tree analysis indicated that a combination of Dfast (Dfast < 13.36 × 10^−3^ mm^2^/s), Dslow (Dslow < 1.152 × 10^−3^ mm^2^/s), and PF (PF < 12.55%), effectively distinguished healthy individuals from all fibrotic livers (F1–4), achieving AUC of 0.986 in logistic regression. The study concluded that a combination of Dfast, Dslow and PF shows the potential of IVIM to detect early-stage liver fibrosis.
Lesheng Huang et al. [27]	N = 145 subjects (48 healthy volunteers, 59 early liver fibrosis patients, and 38 advanced liver fibrosis patients) Prospective	To assess the diagnostic effectiveness of metrics obtained from DWI, IVIM, and DKI for the purpose of staging liver fibrosis, emphasizing robust inter-examiner reliability as a guiding principle	In all study groups, the inter-examiner reliability of the parameters DDC, D, and particularly D* was low was found. Parameters from DTI, DKI, and DWI displayed good to excellent reliability; nonetheless, the majority of DKI, DTI, and DKI parameters did not exhibit notable variances among the cohorts under investigation The study findings reported that the specificity and sensitivity of the models distinguishing healthy volunteers from early liver fibrosis and those differentiating early liver fibrosis from advanced liver fibrosis were poor.
Li Yang [40]	N = 81 patients chronic liver disease Prospective	Compared the efficacy of KDI to conventional DWI among these patients	The kurtosis model does not provide any additional benefit over the conventional monoexponentially model.
Yoshimaru et al. [48]	N = 67 Prospective	To assess the potential of DKI analysis using the breath-hold technique for evaluating liver fibrosis	The DKI cutoff values for assessing F0, ≥F1, ≥F2, and F4 were 0.923, 0.955, and 1.11, respectively. ADC values did not demonstrate a correlation with the severity of liver fibrosis staging
Shuangshuang Xie et al. [50]	N = 45 patients (*n* = 25 mild (S1) or *n* = 20 substantial (S2)) and 27 healthy controls Prospective	To assess the effectiveness of DKI in distinguishing between healthy controls and patients with S1 and S2 fibrosis, and to compare its diagnostic accuracy to traditional DWI	Strong agreement was found in the inter-observer reproducibility of the ADC, MK, and MD measures (ICC = 0.912, 0.908, and 0.894, respectively). Both ADC (*p* = 0.013) and MD (*p* < 0.001) values showed a decreasing trend with increasing fibrotic stage, showing notable distinctions between healthy participants and individuals with S1 and S2 fibrosis. Furthermore, there were statistically significant differences in MD values between S0 and S1 (*p* = 0.028) as well as S0 and S2 (*p* = 0.005), with no notable difference observed between S1 and S2 (*p* = 0.452). There was a negative association between the stages of fibrosis and the ADC as well as MD values (rs = 0.668, −0.341; *p* < 0.01), while MK levels did not show a significant connection with fibrosis stages (rs = 0.180; *p* = 0.13).

**Table 4:** Summary of radiomics analysis of DWI images studies to evaluate liver fibrosis.

Ref.	Study Design	Target	Aim	Findings
Qiu et al. [56]	Retrospective	369 participants with liver fibrosis (*n* = 108) and early-stage cirrhosis (*n* = 116) and control (*n* = 145)	To develop a radiomics model to detect early-stage cirrhosis and liver fibrosis	Radiomics analysis of DW images can accurately identify early-stage cirrhosis and liver fibrosis, with AUC values ranging from 0.944 to 0.973
Gotta et al. [58]	Prospective	79 participants (no liver fibrosis *n* = 31 participants and 48 with histologically proven fibrosis.	To develop a reliable grading system for liver fibrosis that does not require invasive procedures including ADC	The combination of ADC with radiomics did not reliably improve the predictive accuracy for grading fibrosis. Although there was a tendency for an increase in AUC for fibrosis grade 3 with the inclusion of ADC, the variations observed across all grades were not statistically significant

## Data Availability

Not applicable.

## References

[1] Parola M., Pinzani M. (2019). Liver fibrosis: Pathophysiology, pathogenetic targets and clinical issues. Mol. Asp. Med..

[2] Berumen J., Baglieri J., Kisseleva T., Mekeel K. (2021). Liver fibrosis: Pathophysiology and clinical implications. WIREs Mech. Dis..

[3] Goodman Z.D. (2007). Grading and staging systems for inflammation and fibrosis in chronic liver diseases. J. Hepatol..

[4] Khalifa A., Rockey D.C. (2020). The utility of liver biopsy in 2020. Curr. Opin. Gastroenterol..

[5] Maino C., Vernccio F., Cannella R., Cristoferi L., Franco P.N., Carbone M., Cortese F., Faletti R., De Bernardi E., Inchingolo R. (2024). Non-invasive imaging biomarkers in chronic liver disease. Eur. J. Radiol..

[6] Jiang H., Chen J., Gao R., Huang Z., Wu M., Song B. (2017). Liver fibrosis staging with diffusion-weighted imaging: A systematic review and meta-analysis. Abdom. Radiol..

[7] Lincke T., Zech C.J. (2017). Liver metastases: Detection and staging. Eur. J. Radiol..

[8] Petitclerc L., Gilbert G., Nguyen B.N., Tang A. (2017). Liver fibrosis quantification by magnetic resonance imaging. Top. Magn. Reson. Imaging.

[9] Wang Y., Jin Z.-Y. (2019). Radiomics approaches in gastric cancer: A frontier in clinical decision making. Chin. Med. J..

[10] Liu Z., Wang S., Dong D., Wei J., Fang C., Zhou X., Sun K., Li L., Li B., Wang M. (2019). The applications of radiomics in precision diagnosis and treatment of oncology: Opportunities and challenges. Theranostics.

[11] Zhang Y.-P., Zhang X.-Y., Cheng Y.-T., Li B., Teng X.-Z., Zhang J., Lam S., Zhou T., Ma Z.-R., Sheng J.-B. (2023). Artificial intelligence-driven radiomics study in cancer: The role of feature engineering and modeling. Mil. Med. Res..

[12] Neisius U., El-Rewaidy H., Kucukseymen S., Tsao C.W., Mancio J., Nakamori S., Manning W.J., Nezafat R. (2020). Texture signatures of native myocardial T1 as novel imaging markers for identification of hypertrophic cardiomyopathy patients without scar. J. Magn. Reson. Imaging.

[13] Chea P., Mandell J.C. (2020). Current applications and future directions of deep learning in musculoskeletal radiology. Skelet. Radiol..

[14] Alyami A.S. (2023). The role of radiomics in fibrosis Crohn’s disease: A review. Diagnostics.

[15] Guan Y., Li W., Jiang Z., Chen Y., Liu S., He J., Zhou Z., Ge Y. (2016). Whole-lesion apparent diffusion coefficient-based entropy-related parameters for characterizing cervical cancers: Initial findings. Acad. Radiol..

[16] Corino V.D., Montin E., Messina A., Casali P.G., Gronchi A., Marchianò A., Mainardi L.T. (2018). Radiomic analysis of soft tissues sarcomas can distinguish intermediate from high-grade lesions. J. Magn. Reson. Imaging.

[17] Kocak B., Baessler B., Cuocolo R., Mercaldo N., dos Santos D.P. (2023). Trends and statistics of artificial intelligence and radiomics research in Radiology, Nuclear Medicine, and Medical Imaging: Bibliometric analysis. Eur. Radiol..

[18] Venkatesh S.K., Torbenson M.S. (2022). Liver fibrosis quantification. Abdom. Radiol..

[19] De K. (2005). Nonalcoholic Steatohepatitis Clinical Research Network. Design and validation of a histological scoring system for nonalcoholic fatty liver disease. Hepatology.

[20] Ishak K., Baptista A., Bianchi L., Callea F., De Groote J., Gudat F., Denk H., Desmet V., Korb G., MacSween R.N. (1995). Histological grading and staging of chronic hepatitis. J. Hepatol..

[21] Almpanis Z., Demonakou M., Tiniakos D. (2016). Evaluation of liver fibrosis: “Something old, something new…”. Ann. Gastroenterol. Q. Publ. Hell. Soc. Gastroenterol..

[22] Nallagangula K.S., Nagaraj S.K., Venkataswamy L., Chandrappa M. (2018). Liver fibrosis: A compilation on the biomarkers status and their significance during disease progression. Future Sci. OA.

[23] Gourtsoyianni S., Santinha J., Matos C., Papanikolaou N. (2020). Diffusion-weighted imaging and texture analysis: Current role for diffuse liver disease. Abdom. Radiol..

[24] Le Bihan D., Breton E., Lallemand D., Grenier P., Cabanis E., Laval-Jeantet M. (1986). MR imaging of intravoxel incoherent motions: Application to diffusion and perfusion in neurologic disorders. Radiology.

[25] Li Y.T., Cercueil J.-P., Yuan J., Chen W., Loffroy R., Wáng Y.X.J. (2017). Liver intravoxel incoherent motion (IVIM) magnetic resonance imaging: A comprehensive review of published data on normal values and applications for fibrosis and tumor evaluation. Quant. Imaging Med. Surg..

[26] Iima M. (2021). Perfusion-driven intravoxel incoherent motion (IVIM) MRI in oncology: Applications, challenges, and future trends. Magn. Reson. Med. Sci..

[27] Huang L., Wei Q., Peng H., Zhang W., Tang J., Liu T. (2024). Monoexponential and advanced diffusion-weighted imaging for hepatic fibrosis staging based on high inter-examiner reliability. Saudi Med. J..

[28] Seo N., Chung Y.E., Park Y.N., Kim E., Hwang J., Kim M.-J. (2018). Liver fibrosis: Stretched exponential model outperforms mono-exponential and bi-exponential models of diffusion-weighted MRI. Eur. Radiol..

[29] Park J.H., Seo N., Chung Y.E., Kim S.U., Park Y.N., Choi J.-Y., Park M.-S., Kim M.-J. (2021). Noninvasive evaluation of liver fibrosis: Comparison of the stretched exponential diffusion-weighted model to other diffusion-weighted MRI models and transient elastography. Eur. Radiol..

[30] Jensen J.H., Helpern J.A., Ramani A., Lu H., Kaczynski K. (2005). Diffusional kurtosis imaging: The quantification of non-gaussian water diffusion by means of magnetic resonance imaging. Magn. Reson. Med..

[31] Lu H., Jensen J.H., Ramani A., Helpern J.A. (2006). Three-dimensional characterization of non-gaussian water diffusion in humans using diffusion kurtosis imaging. NMR Biomed..

[32] Huang M., Lu X., Wang X., Shu J. (2020). Diffusion tensor imaging quantifying the severity of chronic hepatitis in rats. BMC Med. Imaging.

[33] Shenoy-Bhangle A., Baliyan V., Kordbacheh H., Guimaraes A.R., Kambadakone A. (2017). Diffusion weighted magnetic resonance imaging of liver: Principles, clinical applications and recent updates. World J. Hepatol..

[34] Mathew R.P., Venkatesh S.K. (2018). Imaging of hepatic fibrosis. Curr. Gastroenterol. Rep..

[35] Kromrey M.-L., Le Bihan D., Ichikawa S., Motosugi U. (2020). Diffusion-weighted MRI-based virtual elastography for the assessment of liver fibrosis. Radiology.

[36] Wang J., Zhou X., Yao M., Tan W., Zhan S., Liu K., Feng Z., Yan H., Dai Y., Yuan J. (2024). Comparison and optimization of b value combinations for diffusion-weighted imaging in discriminating hepatic fibrosis. Abdom. Radiol..

[37] Li T., Che-Nordin N., Wáng Y.X.J., Rong P.-F., Qiu S.-W., Zhang S.-W., Zhang P., Jiang Y.-F., Chevallier O., Zhao F. (2019). Intravoxel incoherent motion derived liver perfusion/diffusion readouts can be reliable biomarker for the detection of viral hepatitis B induced liver fibrosis. Quant. Imaging Med. Surg..

[38] Yoon J.H., Lee J.M., Lee K.B., Kim D., Kabasawa H., Han J.K. (2019). Comparison of monoexponential, intravoxel incoherent motion diffusion-weighted imaging and diffusion kurtosis imaging for assessment of hepatic fibrosis. Acta Radiol..

[39] Zawada E., Serafin Z., Dybowska D., Halota W., Wypych A., Nadolska K., Rusak G. (2019). Monoexponential and biexponential fitting of diffusional magnetic resonance imaging signal analysis for prediction of liver fibrosis severity. J. Comput. Assist. Tomogr..

[40] Yang L., Rao S., Wang W., Chen C., Ding Y., Yang C., Grimm R., Yan X., Fu C., Zeng M. (2018). Staging liver fibrosis with DWI: Is there an added value for diffusion kurtosis imaging?. Eur. Radiol..

[41] Zheng Y., Xu Y.S., Liu Z., Liu H.F., Zhai Y.N., Mao X.R., Lei J.Q. (2020). Whole-liver apparent diffusion coefficient histogram analysis for the diagnosis and staging of liver fibrosis. J. Magn. Reson. Imaging.

[42] Chen F., Chen Y.-L., Chen T.-W., Li R., Pu Y., Zhang X.-M., Li H.-J., Tang S., Cao J.-M., Yang J.-Q. (2020). Liver lobe based intravoxel incoherent motion diffusion weighted imaging in hepatitis B related cirrhosis: Association with child-pugh class and esophageal and gastric fundic varices. Medicine.

[43] Tosun M., Onal T., Uslu H., Alparslan B., Akhan S.Ç. (2020). Intravoxel incoherent motion imaging for diagnosing and staging the liver fibrosis and inflammation. Abdom. Radiol..

[44] Wáng Y.X.J., Deng M., Li Y.T., Huang H., Leung J.C.S., Chen W., Lu P.-X. (2018). A combined use of intravoxel incoherent motion MRI parameters can differentiate early-stage hepatitis-b fibrotic livers from healthy livers. SLAS Technol. Transl. Life Sci. Innov..

[45] Ding L., Xiao L., Lin X., Xiong C., Lin L., Chen S. (2018). Intravoxel Incoherent Motion (IVIM) Diffusion-Weighted Imaging (DWI) in Patients with Liver Dysfunction of Chronic Viral Hepatitis: Segmental Heterogeneity and Relationship with Child-Turcotte-Pugh Class at 3 Tesla. Gastroenterol. Res. Pract..

[46] Hu F., Yang R., Huang Z., Wang M., Zhang H., Yan X., Song B. (2017). Liver fibrosis: In vivo evaluation using intravoxel incoherent motion-derived histogram metrics with histopathologic findings at 3.0 T. Abdom. Radiol..

[47] Ren H., Xu H., Yang D., Tong X., Zhao X., Wang Q., Sun Y., Ou X., Jia J., You H. (2024). Intravoxel incoherent motion assessment of liver fibrosis staging in MASLD. Abdom. Radiol..

[48] Yoshimaru D., Miyati T., Suzuki Y., Hamada Y., Mogi N., Funaki A., Tabata A., Masunaga A., Shimada M., Tobari M. (2018). Diffusion kurtosis imaging with the breath-hold technique for staging hepatic fibrosis: A preliminary study. Magn. Reson. Imaging.

[49] Fu F., Li X., Chen C., Bai Y., Liu Q., Shi D., Sang J., Wang K., Wang M. (2020). Non-invasive assessment of hepatic fibrosis: Comparison of MR elastography to transient elastography and intravoxel incoherent motion diffusion-weighted MRI. Abdom. Radiol..

[50] Xie S., Li Q., Cheng Y., Zhou L., Xia S., Li J., Shen W. (2020). Differentiating mild and substantial hepatic fibrosis from healthy controls: A comparison of diffusion kurtosis imaging and conventional diffusion-weighted imaging. Acta Radiol..

[51] Ren H., Liu Y., Lu J., An W., Wang W., Yan T., Li Y., Dong J., Cai J. (2021). Evaluating the clinical value of MRI multi-model diffusion-weighted imaging on liver fibrosis in chronic hepatitis B patients. Abdom. Radiol..

[52] Jiang Y.-L., Li J., Zhang P.-F., Fan F.-X., Zou J., Yang P., Wang P.-F., Wang S.-Y., Zhang J. (2024). Staging liver fibrosis with various diffusion-weighted magnetic resonance imaging models. World J. Gastroenterol..

[53] Huang H., Che-Nordin N., Wang L.-F., Xiao B.-H., Chevallier O., Yun Y.-X., Guo S.-W., Wáng Y.X.J. (2019). High performance of intravoxel incoherent motion diffusion MRI in detecting viral hepatitis-b induced liver fibrosis. Ann. Transl. Med..

[54] Sollini M., Antunovic L., Chiti A., Kirienko M. (2019). Towards clinical application of image mining: A systematic review on artificial intelligence and radiomics. Eur. J. Nucl. Med. Mol. Imaging.

[55] Zheng W., Guo W., Xiong M., Chen X., Gao L., Song Y., Cao D. (2023). Clinic-radiological features and radiomics signatures based on Gd-BOPTA-enhanced MRI for predicting advanced liver fibrosis. Eur. Radiol..

[56] Qiu Q.-T., Zhang J., Duan J.-H., Wu S.-Z., Ding J.-L., Yin Y. (2020). Development and validation of radiomics model built by incorporating machine learning for identifying liver fibrosis and early-stage cirrhosis. Chin. Med. J..

[57] Xiao L., Zhao H., Liu S., Dong W., Gao Y., Wang L., Huang B., Li Z. (2024). Staging liver fibrosis: Comparison of radiomics model and fusion model based on multiparametric MRI in patients with chronic liver disease. Abdom. Radiol..

[58] Gotta J., Gruenewald L.D., Reschke P., Booz C., Mahmoudi S., Stieltjes B., Choi M.H., D’Angelo T., Bernatz S., Vogl T.J. (2025). Noninvasive Grading of Liver Fibrosis Based on Texture Analysis From MRI-Derived Radiomics. NMR Biomed..

